# Tocilizumab in patients with anti-TNF refractory juvenile idiopathic arthritis-associated uveitis (APTITUDE): a multicentre, single-arm, phase 2 trial

**DOI:** 10.1016/S2665-9913(20)30008-4

**Published:** 2020-02-07

**Authors:** Athimalaipet V Ramanan, Andrew D Dick, Catherine Guly, Andrew McKay, Ashley P Jones, Ben Hardwick, Richard W J Lee, Matthew Smyth, Thomas Jaki, Michael W Beresford, APTITUDE Trial Management Group

**Affiliations:** a University Hospitals Bristol NHS Foundation Trust and Bristol Medical School, University of Bristol, Bristol, UK; b Bristol Eye Hospital, Bristol, UK; c School of Clinical Sciences, University of Bristol, Bristol, UK; d UCL Institute of Ophthalmology and National Institute for Health Research Biomedical Research Centre at Moorfields Eye Hospital, London, UK; e University College London Institute of Ophthalmology, London, UK; f Liverpool Clinical Trials Centre, Department of Biostatistics, University of Liverpool, Liverpool, UK; g Mathematics and Statistics, Lancaster University, Lancaster, UK; h Department of Women's and Children's Health, Institute of Translational Medicine, University of Liverpool, Liverpool, UK; i Department of Paediatric Rheumatology, Alder Hey Children's NHS Foundation Trust, Liverpool, UK

## Abstract

**Background:**

Uveitis associated with juvenile idiopathic arthritis is a cause of major ocular morbidity. A substantial proportion of children are refractory to systemic methotrexate and TNF inhibitors. Our aim was to study the safety and efficacy of tocilizumab in children with juvenile idiopathic arthritis-associated uveitis refractory to both methotrexate and TNF inhibitors.

**Methods:**

This multicentre, single-arm, phase 2 trial was done following a Simon's two-stage design at seven tertiary hospital sites in the UK. Patients aged 2–18 years with active juvenile idiopathic arthritis-associated uveitis were eligible. All patients had been on a stable dose of methotrexate for at least 12 weeks and had not responded to treatment with a TNF inhibitor. Patients weighing 30 kg or more were treated with 162 mg subcutaneous tocilizumab every 2 weeks for 24 weeks, and participants weighing less than 30 kg were treated with 162 mg every 3 weeks for 24 weeks. The primary outcome was treatment response defined as a two-step decrease, or decrease to zero, from baseline in the level of inflammation (anterior chamber cells) at week 12, per the standardisation of uveitis nomenclature criteria. A phase 3 trial would be justified if more than seven patients responded to treatment. An interim analysis was planned to assess whether the trial would be stopped for futility, with futility defined as two or fewer treatment responses among ten participants. Adverse events were collected up to 30 calendar days after treatment cessation. The primary analysis was done in the intention-to-treat population and the safety analysis was done in all patients who started the treatment. This trial is registered with the International Standard Randomised Controlled Trial Number registry (ISRCTN95363507) and EU Clinical Trials Register (EudraCT 2015-001323-23).

**Findings:**

22 participants were enrolled to the trial between Dec 3, 2015, and March 9, 2018, and 21 participants received treatment. One participant was found to be ineligible immediately after enrolment and was therefore withdrawn. Seven of 21 (median unbiased estimate of proportion 34% [95% CI 25–57]) responded to treatment (p=0·11). Safety results were consistent with the known safety profile of tocilizumab.

**Interpretation:**

The primary endpoint was not met, and thus the results do not support a phase 3 trial of tocilizumab in patients with juvenile idiopathic arthritis-associated uveitis. Importantly, data on the use of tocilizumab in clinical practice is now captured in national registries. Despite this trial not meeting the threshold required to justify a larger phase 3 trial, several patients responded to treatment; as such, tocilzumab might still be a therapeutic option in some children with uveitis refractory to anti-TNF drugs, given the absence of other treatment options.

**Funding:**

Versus Arthritis and the National Institute for Health Research Clinical Research Network: Children.

## Introduction

Juvenile idiopathic arthritis is an inflammatory arthritis that affects one in 1000 children. Children with juvenile idiopathic arthritis are also at risk of uveitis, an inflammation of the uvea in the eye. Up to 80% of all paediatric uveitis is secondary to juvenile idiopathic arthritis.^1^, ^2^ The development of juvenile idiopathic arthritis with uveitis is associated with early onset of arthritis, an oligoarticular pattern of arthritis, and presence of antinuclear antibodies.^3^

Children with moderate to severe uveitis can be refractory to methotrexate.^4^, ^5^, ^6^, ^7^, ^8^ In such patients, monoclonal TNF inhibitors, including adalimumab, are often effective.^9^, ^10^, ^11^, ^12^ However, 30–40%^13^ of patients are refractory to both methotrexate and TNF inhibitors and are therefore at great risk of significant ocular complications and blindness.

In patients with severe disease that does not respond to methotrexate and anti-TNF drugs, strong evidence supports the approach of targeting interleukin-6 (IL-6) in the disease pathogenesis.^14^, ^15^, ^16^, ^17^, ^18^ Therefore, a phase 2 trial of the potential efficacy, safety, and tolerability of the IL-6 receptor inhibitor tocilizumab was done. In arthritis, IL-6 causes tiredness, anaemia, and inflammation, as well as damage to bones, cartilage, and tissue; tocilizumab reduces these effects.^19^ Previous studies looking at the effect of tocilizumab in children have been done looking at rheumatological examinations only.^20^ However, in a trial of tocilizumab in children with the systemic form of juvenile idiopathic arthritis who are unresponsive to methotrexate, patients responded dramatically to treatment in a short time span.^20^ As a result, tocilizumab became the first drug licenced for use in juvenile idiopathic arthritis in 50 years; tocilizumab also obtained National Institute for Health and Care Excellence approval for this indication in 2011. An ongoing clinical trial is testing tocilizumab in patients with polyarticular forms of juvenile idiopathic arthritis with good effect (NCT02165345). However, patients with juvenile idiopathic arthritis-associated uveitis have been excluded from these clinical trials, so the efficacy of tocilizumab in these patients is unclear. Therefore, in the APTITUDE trial, we aimed to assess the safety and efficacy of tocilizumab in this paediatric population.

**Research in context**

**Evidence before this study**

We reviewed an evidence synthesis review update in April, 2013, and before grant submission, prepared by Arthritis Research UK (now Versus Arthritis). The object of this report was to highlight recently completed and ongoing clinical trials in Paediatric Rheumatology. We found no studies related to tocilizumab and juvenile idiopathic arthritis-associated uveitis. We also searched ClinicalTrials.gov using the search terms “uveitis” and “tocilizumab”. We found one open-label trial aiming to assess tocilizumab treatment in six patients with juvenile idiopathic arthritis-associated uveitis. The efficacy of tocilizumab in uveitis and ophthalmology outcomes had not been assessed before that study. However, the rationale for anti-IL-6 therapy is strong—in arthritis, IL-6 causes tiredness, anaemia, and inflammation and damage to bones, cartilage, and tissue. Tocilizumab blocks IL-6, reducing the symptoms. Hence, a phase 2 study is needed to give early indications of the clinical effectiveness of tocilizumab in combination with methotrexate and to decide whether further research is justified. Previous studies investigating the effect of tocilizumab in paediatric arthritis have excluded patients with uveitis. However, a study by Muselier and colleagues on tocilizumab in uveitis in adults showed its potential role for refractory disease. A previous systematic search by Adán and colleagues of existing data found only a couple of case reports.

**Added value of this study**

To our knowledge, this is first trial looking at efficacy of tocilizumab in juvenile idiopathic arthritis-associated uveitis. This study is able to give early indications of the potential clinical effectiveness of tocilizumab in combination with methotrexate for the treatment of children with refractory juvenile idiopathic arthritis-associated uveitis.

**Implications of all the available evidence**

This study provides evidence that tocilizumab might be a useful adjunctive therapeutic option for children with uveitis refractory to anti-TNF treatments. This study also provides evidence of efficacy in macular oedema associated with juvenile idiopathic arthritis uveitis, as reported in previous studies.

## Methods

### Study design

APTITUDE was a multicentre, single-arm, phase 2 trial that was done at seven tertiary hospital sites in the UK following a Simon's two-stage design,^21^ in which a small group of participants are recruited in the first stage, and the recruitment of another group of participants in stage 2 only commences if an adequate number of responses have been observed in the first stage (appendix p 1).

Ethical approval for the trial was provided by the National Research Ethics Service Committee London—South East on July 3, 2015 (reference number 15/LO/0771).

The protocol and statistical analysis plan are available online.

### Patients

Children and young people aged 2–18 years of age who had active juvenile idiopathic arthritis-associated uveitis were eligible to take part in the trial. Active uveitis was defined based on the standardisation of uveitis nomenclature (SUN) criteria^22^ as two or more readings of cellular infiltrate in anterior chamber cells of grade 1+ or greater (possible scores are 0, 0·5+, 1+, 2+, 3+, and 4+) during the 6 weeks preceding screening. Participants must have had an inadequate treatment response with at least one anti-TNF drug and have been on at least one anti-TNF drug (regardless of dose) for at least 12 weeks at any time before enrolment in the trial. They must have been on methotrexate for at least 12 weeks with a stable dose for 4 weeks before screening, without adequate response. Key exclusion criteria were previous exposure to tocilizumab; previous exposure in a clinical trial to another medicinal product such that the estimated level of the drug in the patient's blood was more than that predicted by 4 weeks or five half-lives of the drug (whichever was longer); receipt of more than six topical glucocorticoid drops per eye per day at time of enrolment; and receipt of prednisone (or the equivalent) at a dose exceeding 0·2 mg/kg bodyweight per day. Patients who did not pass screening were able to be re-screened after a minimum of 1 week after their last screening. Full exclusion criteria are in the appendix (p 3). Each parent or guardian provided written informed consent. Each child gave assent when appropriate.

### Procedures

Patients received tocilizumab dosed according to bodyweight, with patients weighing 30 kg or more given 162 mg of subcutaneous tocilizumab every 2 weeks and patients weighing less than 30 kg given 162 mg of subcutaneous tocilizumab every 3 weeks for 24 weeks. Patients weighing less than 30 kg were given a maximum of nine injections and patients weighing 30 kg or more were given a maximum of 13 injections. Injections were administered at hospital or by self administration at home depending on patient preference. Patients who missed two consecutive doses or three doses in total of tocilizumab injection ceased trial treatment and were recorded as a withdrawal from treatment. Treatment compliance was measured using accountability logs and participant diaries. All participants were treated up to a maximum of 24 weeks and then followed up after treatment for 12 weeks and assessed per the trial assessments (appendix p 2). All patients continued on methotrexate throughout trial participation.

Adverse events were collected up to 30 calendar days after cessation of treatment. Patients who did not achieve treatment response stopped treatment and proceeded to follow-up.

### Outcomes

The primary outcome was response to treatment, defined according to SUN criteria^22^ as a two-step decrease in score in the level of inflammation (anterior chamber cells) or decrease to zero between baseline and 12 weeks of treatment. Secondary outcomes included safety and tolerability of tocilizumab; compliance; corticosteroid use; optic and ocular outcomes; quality of life; American College of Rheumatology (ACR) pediatric 30, ACR pediatric 50, ACR pediatric 70, ACR pediatric 90, and ACR pediatric 100; numbers of participants with changes in biologic or disease-modifying anti-rheumatic drugs; numbers of patients with arthritis flares; and juvenile arthritis disease activity score (JADAS). Details are in the appendix (pp 4–5).

### Statistical analysis

The trial was done following a Simon's two-stage design.^21^ The null hypothesis (response 20% or lower) reflected a response rate of no clinical benefit whereas the alternative hypothesis (response at least 50%) reflects a desired response. If the true success probability was 20%, then the probability of success in further study of tocilizumab would be less than 5% (ie, falsely pursuing a non-promising therapy). If the true success probability was 50% or more, then further study of tocilizumab in a phase 3 trial would be recommended, with a probability greater than 90% of showing a therapeutic effect of the drug (ie, correctly pursuing a promising therapy).

The interim and final sample sizes and the critical values for abandoning tocilizumab at each stage were chosen a priori as follows: the interim analysis sample size was ten patients, and the analysis was done after ten patients had provided primary outcome data at the 12-week visit. Based on a critical interim value of two treatment responses (ie, if there were two or fewer treatment responses then the trial would be stopped for futility), a sample size of 22 patients was needed for the full analysis, with a critical value of seven treatment responses (ie, if there were seven or fewer treatment responses, then it would be concluded that the further study of tocilizumab should be abandoned). If further study of the drug is not abandoned at either the interim or the final analysis, then a recommendation would be made to conduct a comparative, randomised phase 3 trial. The interim analysis was reviewed by the independent safety monitoring committee, who made recommendations to the trial steering committee whether to continue the trial or terminate it for futility.

Analyses was done according to the predefined statistical analysis plan and used the principle of intention to treat. If consent to treatment was withdrawn but the participant agreed to remain in the study for follow-up, the participant was followed up until 12 weeks after ceasing trial treatment. If the participant decided to withdraw consent completely, the reasons for withdrawal of consent were recorded (if possible) and reported.

For the primary outcome, the point estimate, CI, and p value were computed using the method described by Jovic and Whitehead.^23^ Prespecified sensitivity analyses tested the effects of missing data, participants who stopped their intervention early, and those who had been incorrectly identified as having treatment response. Trial oversight was provided by an independent data and safety monitoring committee and a trial steering committee.

All analyses were done with SAS, version 9.3 or above. The trial was registered on the International Standard Randomised Controlled Trial Number registry (ISRCTN95363507) on June 10, 2015, and EU Clinical Trials Register on July 3, 2015 (EudraCT 2015-001323-23).

### Role of the funding source

The funder of the study had no role in study design, data collection, data analysis, data interpretation, or writing of the report. The corresponding author had full access to all the data in the study and had final responsibility for the decision to submit for publication.

## Results

Patients were recruited for the trial between Dec 3, 2015, and March 9, 2018. 44 patients were screened (58 screening events) at seven tertiary hospital sites in the UK (appendix p 20). 24 patients (32 screenings) did not meet the inclusion criteria and 26 patients met the inclusion criteria (six of these patients were deemed eligible after not meeting inclusion criteria at an earlier visit; figure). The main reasons for ineligibility were absence of active anterior uveitis as defined in the protocol (17 [53%] of 32 screening events) and presence of clinically significant deviations in laboratory parameters (four [13%]; appendix p 6). Four patients did not consent to take part in the trial. 22 participants were enrolled in the trial.

**Figure:**
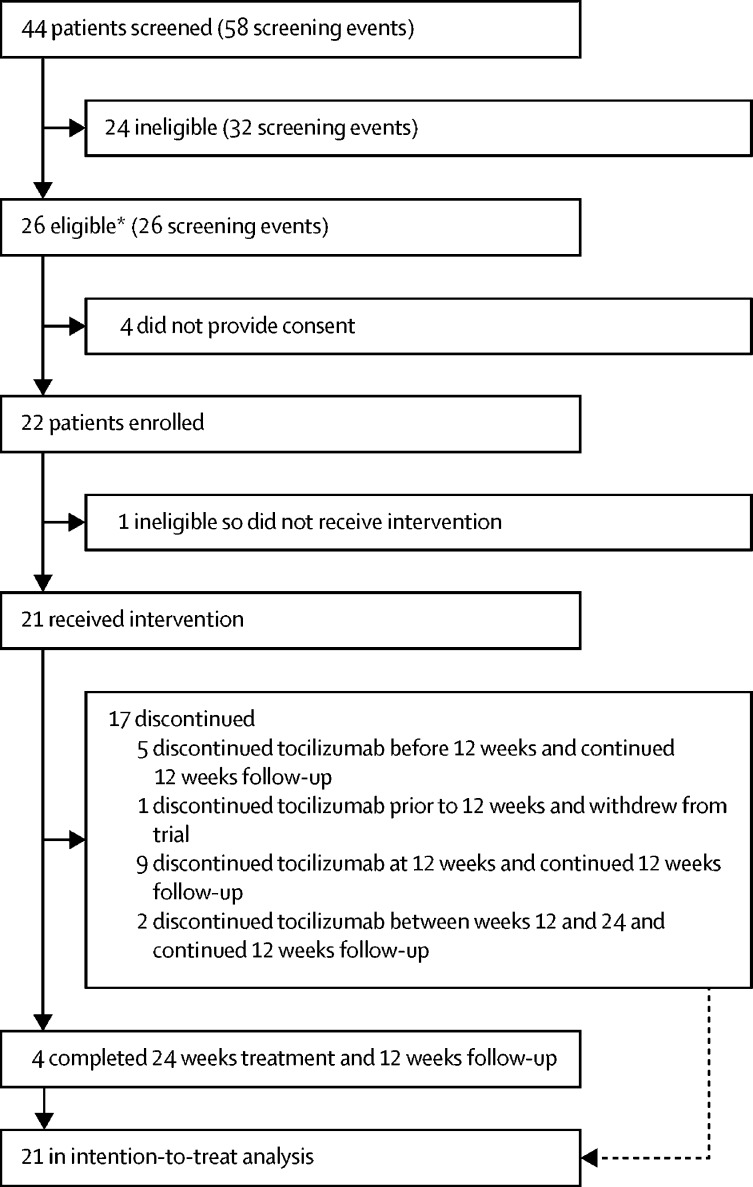
Trial profile *Patients could be screened multiple times; six of these patients were ineligible at an earlier visit.

Particpants were aged 5–17 years and 18 (86%) were women (table 1). All participants in the study had taken adalimumab and none had received other TNF inhibitors; additional demographic and baseline clinical information are in the appendix (pp 6–8). Data for the primary outcome were available for all the participants except one, who was found to be ineligible immediately after enrolment; this patient was therefore not included in any of the analyses. 17 (81%) of 21 patients discontinued treatment before 24 weeks, six (29%) discontinued before their 12-week visit, nine (43%) discontinued at 12 weeks, and two (10%) discontinued between weeks 12 and 24 (one because of non-response and the other because of a requirement for a non-permitted medication to treat worsening ocular pressures). Treatment compliance was 92% according to the patient diaries and 80% according to accountability logs.

**Table 1:** Baseline demographic details

	**Tocilizumab (n=21)**
**Number of study eyes**	
Unilateral	13 (62%)
Bilateral	8 (38%)
**Age at enrolment, years**	
Mean (SD)	12·3 (3·5)
Median (IQR)	12·8 (10·4–15·1)
Range	5·4–17·3
**Sex**	
Female	18 (86%)
Male	3 (14%)
**Weight, kg**	
<30	6 (29%)
≥30	15 (71%)

Data are n (%) unless otherwise stated.

The results of the interim analysis were reported to the independent safety monitoring committee in November, 2016. Four (40%) of ten participants responded to treatment, indicating that the trial should continue.

Seven (33%) of 21 participants achieved treatment response at week 12 (table 2). The median unbiased estimate of the proportion of treatment responses was 34% (95% CI 25–57; p=0·11). Of the six patients that discontinued treatment before week 12, one was classified as a treatment response and the other five as non-responders. 13 (62%) of 21 patients were classified as non-responders, eight of whom reached 12 weeks of treatment. Of the six patients who continued treatment after 12 weeks, four (67%) were classed as treatment responders at 24 weeks.

**Table 2:** Responders

	**Eligible eye**	**Response eye**	**Right eye SUN grade**		**Left eye SUN grade**	
			Baseline	12 weeks	Baseline	12 weeks
1	Both	Both	2+	0·5+	2+	0·5+
2	Both	Both	3+	0	3+	0·5+
3	Both	Both	3+	1+	3+	0·5+
4	Left	Left	NA	NA	4+	0·5+
5*	Left	Left	NA	NA	2+	0
6	Both	Both	2+	0·5+	2+	0·5+
7	Right	Right	4+	1+	NA	NA

A response was defined as a two-step decrease in SUN^22^ grade or decrease to 0. NA=not applicable. SUN=standardisation of uveitis nomenclature.

* Withdrew from treatment after the week 8 visit and assessed by committee to be a responder at 8 weeks.

The safety data set consisted of all 21 patients who received at least one dose of the study drug. A total of 175 adverse events were reported in 20 (95%) participants; no serious adverse events were reported during the trial (appendix pp 8–10). 21 adverse events of special interest in seven (33%) patients were collected during the trial (appendix p 10). The most frequent adverse events were injection site reaction (24 events in eight [38%] patients), arthralgia (eight events in four [19%] patients), and headache (eight events in five [24%] patients; table 3). Safety results were consistent with the known safety profile for tocilizumab.

**Table 3:** Grade 1–2 adverse events occurring in at least 10% of patients and all grade 3–5 events (n=21)

	**Grade 1–2 (≥10% patients)**	**Grade 3 (all)**
**Eye disorders**		
Uveitis	2; 2 (10%)	1; 1 (5%)
**Gastrointestinal disorders**		
Vomiting	3; 3 (14%)	0;
**General disorders and administration site conditions**		
Injection site reaction	24; 8 (38%)	0
**Infections and infestations**		
Upper respiratory tract infection	3; 3 (14%)	0
**Investigations**		
Alanine aminotransferase increased	4; 3 (14%)	0
Blood triglycerides increased	3; 3 (14%)	0
Intraocular pressure increased	3; 1 (5%)	1; 1 (5%)
Neutrophil count decreased	4; 3 (14%)	0
**Musculoskeletal and connective tissue disorders**		
Arthralgia	8; 4 (19%)	0
**Nervous system disorders**		
Headache	8; 5 (24%)	0
**Respiratory, thoracic, and mediastinal disorders**		
Cough	7; 5 (24%)	0
Oropharyngeal pain	7; 6 (29%)	0

Data are number of events; number of patients (%). No grade 4 (life threatening) or grade 5 (deaths) events occurred. For adverse events reported as severe, any corresponding mild or moderate event that occurred in less than 10% of patients have also been presented.

Secondary outcome results are in the appendix (pp 10–18). Of 21 participants, four (19%) were receiving oral corticosteroids at baseline. Three of these patients were taking 5 mg per day or more at baseline, and none were able to reduce the dose to less than 5 mg per day (appendix p 10). 20 patients were using topical corticosteroid eye drops at baseline, 18 of whom who were on two or more drops per eye per day (appendix p 11). Three (17%) of these 18 patients were able to reduce use to less than two drops per eye per day. Three (15%) of 20 participants were able to completely stop use of corticosteroid eye drops.

Four (19%) patients had macular oedema at baseline, which resolved after treatment in three patients. Two patients had glaucomatous neuropathy at baseline; this resolved during the course of the trial in one patient and the other patient developed neovascularisation, which subsequently resolved. No participants had complete disease control (as defined by SUN criteria^22^ as zero cells) at week 12 or week 24 with topical treatment and subcutaneous tocilizumab.

Quality of life data as measured by the child health questionnaire and childhood health assessment questionnaire were not clinically significant and arthritis disease activity measures (ACR pediatric and JADAS) scores did not change significantly (appendix pp 14–16).

In a post-hoc analysis, three (19%) patients had a partial response (one-step improvement) at the 24-week visit (two patients had 12 weeks of treatment and one had 11 weeks).

The mean number of corticosteroid drops at baseline was 4·48 drops (SD 3·11), which reduced to 4·33 drops (2·29) at 12 weeks (appendix p 11). Foveal thickness split was also assessed post-hoc and the mean number for best score was 278·6 (SD 67·68) reducing to 257·7 (51·73) at 24 weeks (appendix p 17).

## Discussion

This Simon's two-stage design study of participants with treatment refractory juvenile idiopathic arthritis-associated uveitis did not meet the prespecified criterion (more than seven responses from 21 participants) at 12 weeks to justify a phase 3 trial. A third of the participants (seven [33%] of 21) had a two-step improvement in uveitis assessment at week 12 and a further three (14%) had a one-step improvement at week 24 with tocilizumab. Three of four participants had complete resolution of cystoid macular oedema in response to tocilizumab.

Tocilizumab is effective in polyarticular^20^ and systemic juvenile idiopathic arthritis^24^ as well as rheumatoid arthritis^25^ and giant cell arteritis.^26^ The STOP-Uveitis study,^14^ an open-label study of intravenous tocilizumab in adult patients with posterior segment uveitis, showed a reduction in vitreous haze and macular thickness. Improvements in anterior chamber cell counts and macular thickening were seen in retrospective studies of intravenous tocilizumab in juvenile idiopathic arthritis uveitis and adult uveitis, including adult juvenile idiopathic arthritis.^15^, ^16^, ^17^, ^18^

One study of tocilizumab in juvenile idiopathic arthritis used the juvenile idiopathic arthritis ACR pediatric 30 response at 12–16 weeks as the primary outcome,^20^ which although not directly comparable to uveitis scores, are arguably a less robust response than a two-step decrease on the SUN inflammation score. Studies have also shown progressive improvements in uveitis activity^14^ over 26 weeks and systemic juvenile idiopathic arthritis disease activity over 52 weeks^24^ with tocilizumab. It is possible that the number of responders on tocilizumab would have been higher with a longer duration of treatment, but it is also important to protect against the risks of a potentially ineffective treatment. Rapid disease control is important in uveitis, in which prolonged uveitis activity increases the risk of sight loss.^27^

Subcutaneous tocilizumab was less effective than intravenous tocilizumab for juvenile idiopathic arthritis uveitis in a small case series,^28^ but has shown similar efficacy to intravenous tocilizumab in randomised controlled studies of rheumatoid arthritis.^29^, ^30^ Subcutaneous therapies are more desirable than intravenous infusions for patient convenience, maximising school attendance and using fewer health-care resources. The eye has blood aqueous and blood retinal barriers and so potentially higher doses of drug are required in the eye to gain therapeutic efficacy than at other body sites.^31^

The paucity of clinical trials for paediatric uveitis combined with the availability of potential treatments through use in other rheumatic diseases has led to many immunosuppressive drugs being prescribed for paediatric uveitis and included in treatment guidelines with little evidence.^32^ A strength of this study is the incorporation of the Simon design to identify molecules for further clinical study in patients with paediatric uveitis, with low risk to participants, and to enable the gathering of evidence and publication of results in a more systematic way than in case series and open-label studies. Safety results were consistent with the known safety profile of intravenous tocilizumab, with the exception of injection site reactions.^20^, ^24^

Limitations of this study include the small sample size, absence of a control group, and severity of disease. With respect to severity of disease in this cohort, the patients enrolled displayed moderate anterior chamber inflammation, and in some it was associated with cystoid macular oedema despite anti-TNF and methotrexate therapy. Continuing inflammation in the face of substantial immunomodulatory therapy indicates a cohort of patients with moderate to severe disease overall. The 12-week duration of the primary outcome might have underestimated the treatment efficacy, and no uveitis-specific patient-recorded outcome measures were used. Macular oedema outcomes were limited to presence or absence of oedema and central macular thickness, which might vary on different optical coherence tomography machines.^33^ Additionally, the results are not transferable to other formulations of tocilizumab and therefore the optimal method of administration for juvenile idiopathic arthritis-associated uveitis remains unclear. Finally, in this small study no stratification of molecular signatures was done to identify differences between responders and non-responders.

In conclusion, subcutaneous tocilizumab did not meet its primary endpoint in this Simon design study. Efficacy signals for juvenile idiopathic arthritis uveitis were noted, including reduction of macular oedema, as reported in previous studies, but not at a sufficient level to warrant a phase 3 study. There might be merit in studying intravenous formulations of tocilizumab in a select predefined population of patients.

Although this study used the standard and validated measure of anterior chamber cell activity to assess the extent of inflammation, lower levels of persistent inflammation that are more quantifiable by clinical assessment might be more useful for trials and going forward in clinical practice. Laser flare photometry, for example, was adopted in the Adjuvite study.^34^

For patients with refractory uveitis not responsive to anti-TNF drugs, other therapeutic approaches have been reported anecdotally, including checking for antidrug antibodies, weekly adalimumab abatacept, and JAK-kinase inhibitors.^35^, ^36^, ^37^ Tocilizumab might provide a valuable adjunctive therapeutic option for children with uveitis refractory to anti-TNF, particularly as adalimumab is the only evidence-based and licensed therapy. Although our study did not meet its primary endpoint, this is, to our knowledge, the only prospective study of tocilizumab.

## Data sharing

Anonymised data collected during this trial will be available to access. Proposals should be directed to the corresponding author (AVR) on behalf of the APTITUDE Trial Management Group (avramanan@hotmail.com). Access will be provided to researchers after the proposal has been reviewed and agreed by the trial data sharing committee.
