# Supplementary material for: Tocilizumab in patients with anti-TNF refractory juvenile idiopathic arthritis-associated uveitis (APTITUDE): a multicentre, single-arm, phase 2 trial
Source: Lancet Rheumatol. 2020 Feb 7;2(3):e135–41. doi: 10.1016/S2665-9913(20)30008-4 (PMC7134526; doi:10.1016/S2665-9913(20)30008-4)
Supplement: Supplementary appendix [file mmc1.pdf]

THE LANCET
Rheumatology

Supplementary appendix

This appendix formed part of the original submission and has been peer reviewed. We post it as supplied by the authors.

Supplement to: Ramanan AV, Dick AD, Guly C, et al. Tocilizumab in patients with anti-TNF refractory juvenile idiopathic arthritis-associated uveitis (APTITUDE): a multicentre, single-arm, phase 2 trial. *Lancet Rheumatol* 2019; published online Feb 7. hhttps://doi.org/10.1016/S2665-9913(20)30008-4.

Supplementary Figure 1: Schematic of Study Design

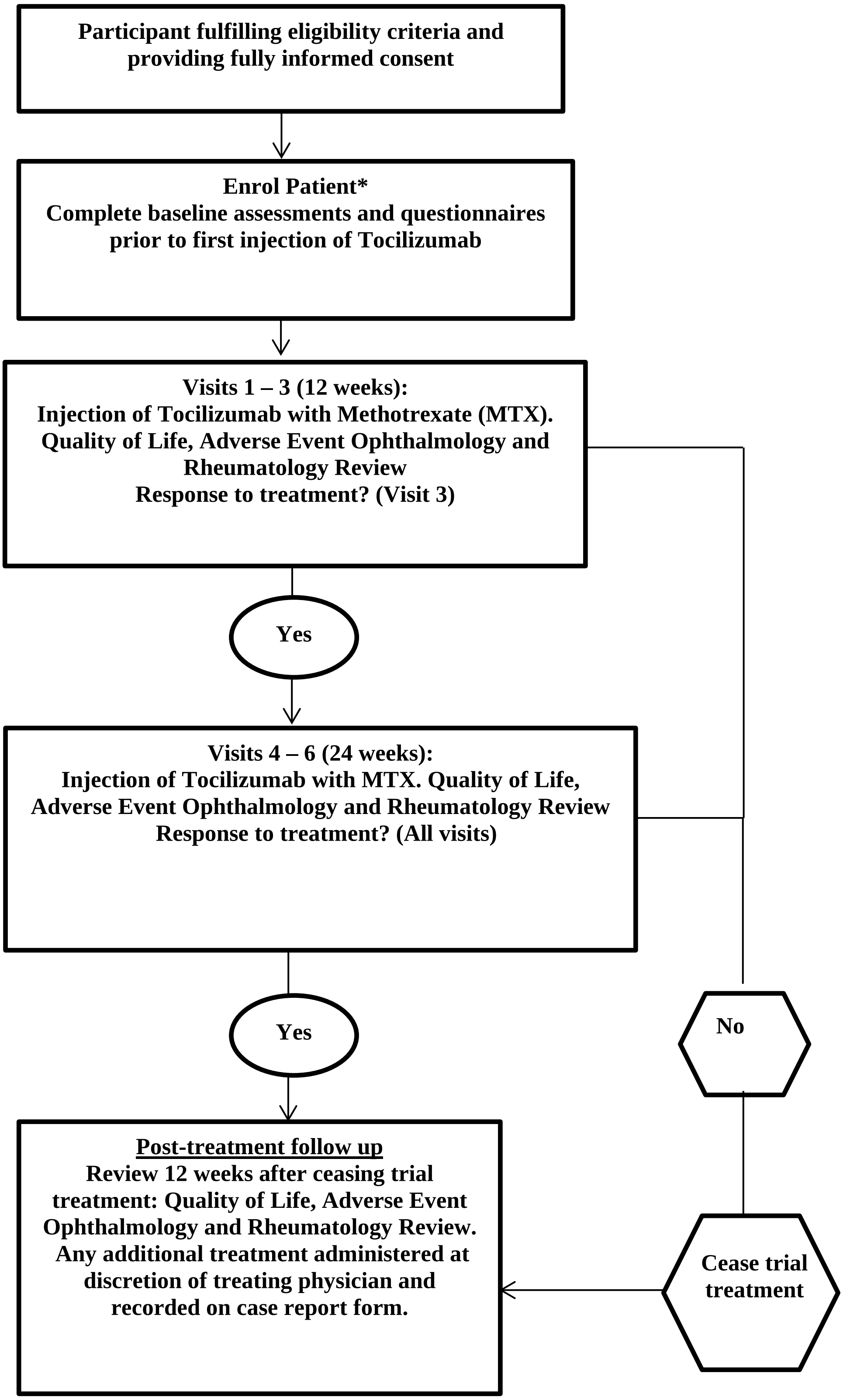

Supplementary Table 1: Study visits and assessments

Assessment (Procedure/ Activity	Screening	Baseline	Visit 1	Visit 2	Visit 3	Visit 4	Visit 5	Visit 6	Visit 7
**Weeks**		0 (-7/+7 days)	4 (-7/+7 days)	8 (-7/+7 days	12 (-7/+7 days)	16 -7/+7 days)	20 (-7/+7 days)	24 (-7/+7 days)	36 (-7/+7 days)
	Screening	Baseline/ Registration	Study Visit	Study Visit	Assessment of endpoints	Study Visit	Study Visit	End of treatment	End of trial
Written and informed consent	X								
Confirm consent	X	X	X	X	X	X	X	X	X
Assessment of eligibility criteria	X	X							
Review of Medical/ Ophthalmic/ Surgical History	X								
Review of concomitant medications	X	X	X	X	X	X	X	X	X
Pregnancy test	X		X	X	X	X	X	X	X
Purified protein derivative Tuberculin Skin Test/ Test latent Tuberculosis as locally performed	X								
Urinalysis	X	X	X	X	X	X	X	X	X
Study intervention (Injection)		X	X	X	X	X	X	X	
Compliance with study intervention		X	X	X	X	X	X	X	
Physical Examination		X	X	X	X	X	X	X	X
Vital signs (heart and respiratory rate and blood pressure)	X		X	X	X	X	X	X	X
Height/ Weight	X	X	X	X	X	X	X	X	X
Dispense treatment diary		X	X	X	X	X	X	X	
Child Health Questionnaire		X	X	X	X	X	X	X	X
Childhood Health Assessment Questionnaire		X	X	X	X	X	X	X	X
Haematological analysis	X	X*	X	X	X	X	X	X	X
Biochemical analysis	X	X*	X	X	X	X	X	X	X
Anti-nuclear antibodies, double stranded deoxyribonucleic acid and extractable nuclear antigens		X						X	
Samples for Biobank		X			X				X
Vision Assessments#	X	X	X	X	X	X	X	X	X
Optical coherence tomography	X		X	X	X	X	X	X	X
Anterior Chamber cells and flare assessment#	X	X	X	X	X	X	X	X	X
Assessment of vitritis and vitreous haze	X		X	X	X	X	X	X	X
Cataract scoring	X		X	X	X	X	X	X	X
Goldmann tonometry or tonopen/ICare	X		X	X	X	X	X	X	X
Standard American College of Rheumatology Pediatric Score Set Outcome Variables		X	X	X	X	X	X	X	X
Tanner Score		X			X			X	X
Assessments of Adverse and Serious Adverse Events		X	X	X	X	X	X		

* Biochemical and Haematological taken at screening can be used at baseline only if taken with 2 weeks of baseline visit.

# Tests do not need to be repeated if screening and baseline visit occurs on the same day.

Supplementary Table 2: Exclusion Criteria

Exclusion Criteria	
Uveitis without a diagnosis of Juvenile Idiopathic Arthritis (JIA) fulfilling International League if Associations for Rheumatology diagnostic criteria for JIA (all subgroups that have uveitis).	Currently on Tocilizumab or has previously received Tocilizumab.
Previous registration into the APTITUDE trial.	Participation in another clinical trial of investigational medicinal product within the last 4 weeks or 5 serum half-lives (whichever is longer) prior to registration.
More than 6 topical steroid eye drops per day per eye at time of registration (dose must be stable for 1 week prior to registration).	For participants on Prednisolone or Prednisolone equivalent, change of dose within 4 weeks prior to registration.
Participants on Prednisone or Prednisone equivalent with a dose >0.2mg/kg per day.	No intraocular injection of disease modification agents including steroids and anti-VEGF within 4 weeks prior to registration.
No intraocular surgery for previous 12 weeks prior to registration or expected/planned for duration of study.	Lack of recovery from recent surgery or surgery within 6 weeks at the time of registration.
Intra-ocular pressure ≥ 25mm Hg at time of registration.	Participants requiring systemic therapy with oral anti-glaucoma medication.
No disease modifying immunosuppressive drugs, other than MTX in the 4 weeks prior to registration	History of active tuberculosis of less than 24 weeks treatment.
Latent Tuberculosis not successfully treated for at least 4 weeks prior to registration (a test for latent tuberculosis infection must be performed within 12 weeks prior to registration).	Auto-immune, rheumatic disease or overlap syndrome other than JIA.
Females who are pregnant, lactating, or intending to become pregnant during trial.	Known human immunodeficiency virus infection or other condition characterized by a compromised immune system.
Any history of alcohol or drug abuse within 24 weeks prior to registration.	Any active acute, sub-acute, chronic, or recurrent bacterial, viral, systemic fungal, infection or any major episode of infection requiring hospitalisation or treatment with IV antibiotics within 4 weeks of registration or treatment with oral antibiotics within 2 weeks of registration.
History of reactivation or new onset of a systemic infection such as herpes zoster or Epstein−Barr virus within 8 weeks prior to registration.	Hepatitis B surface antigen or hepatitis C antibody positivity or chronic viral or autoimmune hepatitis.
History of concurrent serious gastrointestinal disorders.	Evidence of current serious uncontrolled concomitant cardiovascular (including hyperlipidaemia), nervous system, pulmonary (including obstructive pulmonary disease), renal and hepatic disease.
History of or current cancer or lymphoma.	Persistently poorly controlled severe hypertension (>95th percentile for height / age).
Uncontrolled diabetes mellitus.	History of severe allergic or anaphylactic reactions to human, humanized or murine monoclonal antibodies.
No live attenuated vaccines (including seasonal nasal flu vaccine, varicella vaccine for shingles or chickenpox, measles, mumps and rubella (MMR) or MMR varicella, oral polio vaccine and vaccines for yellow fever, measles, mumps or rubella) 4 weeks prior to registration, throughout the duration of the trial and for 8 weeks following the last dose of study drug.	Previous treatment with any cell-depleting therapies, including investigational agents or approved therapies.
Treatment with intravenous gamma globulin or plasmapheresis within 24 weeks of registration	Any previous treatment with alkylating agents such as chlorambucil, or with total lymphoid irradiation
Any significant medical or surgical condition that would risk the participant's safety or their ability to complete the trial	Any joint injections within 4 weeks prior to registration
Any psychological condition that in the opinion of the principal investigator would interfere with safe completion of the trial	Demonstrations of clinically significant deviations from the following laboratory parameters:<br><br>Serum creatinine > 1.5 × the upper limit of normal (ULN) for age and sex<br>Aspartate Aminotransferase Test or ALT > 1.5 × the ULN for age and sex<br>Total bilirubin > 1.3 mg/dL (>23 µmol/L)<br>Platelet count < 150 × 10^3/µL (< 150,000/mm3) (< 150 x 10^9/L)<br>Haemoglobin < 7.0 g/dL (< 4.3 mmol/L)<br>White blood cell count < 5,000/mm^3 (< 5.0 × 10^9/L)<br>Neutrophil count < 2,500/mm^3 (< 2.5 × 10^9/L)

Supplementary Table 3: Trial Secondary Outcomes

Secondary Outcome	Further Details
Adverse Events, Serious Adverse Events and Adverse Events of Special Interest	The following adverse events were of special interest:<br>Serious and/or medically significant infections<br>Myocardial infarction/acute coronary syndrome<br>Gastrointestinal perforations<br>Malignancies<br>Anaphylaxis/Hypersensitivity reactions<br>Demyelinating disorders<br>Stroke<br>Serious and/or medically significant bleeding events<br>Serious and/or medically significant hepatic events
Laboratory parameters	Haematological and biochemical analysis and urinalysis measured during study period and follow up.
Tolerability and compliance	Participant diaries and dosing records determined tolerability and compliance throughout the trial treatment period
Total oral corticosteroid dose	Recorded during study period and follow up
Reduction in and rate of systemic corticosteroid dose from entry dose	Time to reduction to 0mg and <5mg. Recorded during study period and follow up
Topical corticosteroid use (frequency) compared to usage at registration.	Time to reduction to <2 drops and 0 drops. Recorded during study period and follow up
Visual acuity	Measured by Age-appropriate Logarithm of the Minimum Angle of resolution assessment
Number of participants with resolution of associated optic nerve or macular oedema	Assessed by slit lamp bio microscopy or optical coherence tomography
Number of patients who are able to reduce topical or systemic agents for ocular hypertension	

Secondary Outcome	Further Details
Number of participants with disease control	Defined as zero cells, with topical treatment at 12 weeks treatment visit and 24 weeks treatment visit
Number of participants entering disease remission	Defined as zero cells, without topical treatment at 12 and 24 weeks treatment visit
Duration of sustaining inactive disease	Defined as the length of time with zero cells, with or without topical treatment
Failure to reduce eye drops to 2 drops/day by or at the 12 weeks visit	
Quality of Life	Measured using the Childhood Health Questionnaire[18] and Childhood Health Assessment Questionnaire[19]
American College of Rheumatology (ACR) Pedi core set criteria[20]: at ACR30, ACR50, ACR70, ACR90 and ACR100 levels	
Number of participants requiring change in biologic / Disease-modifying anti-rheumatic drugs (DMARDs) therapy due to disease flare of their arthritis or failure to respond to treatment for their arthritis.	
Number of participants undergoing flare of arthritis[21], in remission on and off medication of their Juvenile Idiopathic Arthritis[22] and with minimum disease activity[23].	
Participants score of the Juvenile Arthritis Disease Activity Score (JADAS)[18].	The JADAS comprises four components[18]: physician global assessment of disease activity[19] parent/patient global assessment of well-being[20] active joint count, in 27, 71 or 10 joints; and[21] erythrocyte sedimentation rate.

Supplementary Table 4: Summary of Non-eligibility

Eligibility Criteria	Number of Patients Not Meeting Criteria
Does not have active anterior uveitis (2 readings >1+ or more in the preceding 6 weeks)	17 (53.1%)
Participant has not been on a stable dose of MTX for at least 4 weeks prior to screening visit.	1 (3.1%)
Biologic agent has not been discontinued according to the timelines specified	3 (9.4%)
Not able to commence the trial treatment within 2 weeks of screening	3 (9.4%)
Participation in another clinical trial of Investigation Medicinal Product (IMP) within 4 weeks or 5 serum half- lives	1 (3.1%)
Topical steroid eye drops dose has not been stable for 1 week prior to registration	2 (6.3%)
Patient requires systemic therapy with oral anti-glaucoma medication	1 (3.1%)
History of severe allergic or anaphylactic reactions to human, humanized or murine monoclonal antibodies	2 (6.3%)
Any significant medical or surgical condition that would risk the patient's safety or their ability to complete the trial	1 (3.1%)
Joint injections within 4 weeks prior to registration	1 (3.1%)
Demonstrations of clinically significant deviations in laboratory parameters	4 (12.5%)
Other (No reason provided and needed eye injection)	2 (6.3%)

Supplementary Table 5: Baseline ocular data

Baseline ocular data	Summary	Ocular baseline data – eye level* Tocilizumab (N=29)	Ocular baseline data – best eye** Tocilizumab (N=21)	Ocular baseline data – worst eye*** Tocilizumab (N=21)
**Topical steroid drops**	Missing	1	1	1
	Mean (SD)	2.6 (1.4)	2.6 (1.5)	2.7 (1.4)
	Median (IQR)	3 (1, 4)	3 (1, 4)	3 (1.5, 4)
	(Min, Max)	(0, 5)	(0, 5)	(0, 5)
**Logarithm of the Minimum Angle of Resolution (LogMAR) score**	Mean (SD)	0.1 (0.3)	0.1 (0.2)	0.2 (0.3)
	Median (IQR)	0 (-0.1, 0.2)	0 (-0.1, 0.2)	0.1 (0, 0.2)
	(Min, Max)	(-0.1, 1.2)	(-0.1, 0.5)	(-0.1, 1.2)
**Anterior Chamber (AC) cells Standardization of Uveitis Nomenclature (SUN)**	1+	6 (20.7%)	5 (23.8%)	5 (23.8%)
	2+	9 (31%)	7 (33.3%)	5 (23.8%)
	3+	11 (37.9%)	6 (28.6%)	8 (38.1%)
	4+	3 (10.3%)	3 (14.3%)	3 (14.3%)
**Flare score (SUN)**	0	4 (13.8%)	3 (14.3%)	2 (9.5%)
	1+	21 (72.4%)	15 (71.4%)	16 (76.2%)
	2+	4 (13.8%)	3 (14.3%)	3 (14.3%)
**Lens Opacities Classification System (LOCS) III Grading: Pseudophakic**	No	25 (86.2%)	18 (85.7%)	18 (85.7%)
	Yes	4 (13.8%)	3 (14.3%)	3 (14.3%)
**LOCS III Grading: Nuclear**	Not done	4	3	3
	N0	24 (96%)	17 (94.4%)	17 (94.4%)
	NI	1 (4%)	1 (5.6%)	1 (5.6%)
**LOCS III Grading: Cortical**	Not done	4	3	3
	No Cortical Cataract	24 (96%)	18 (100%)	17 (94.4%)
	CI	1 (4%)	0 (0%)	1 (5.6%)
**LOCS III Grading: Posterior**	Not done	4	3	3
	0	24 (96%)	17 (94.4%)	17 (94.4%)
	PI	1 (4%)	1 (5.6%)	1 (5.6%)

Baseline ocular data	Summary	Ocular baseline data – eye level* Tocilizumab (N=29)	Ocular baseline data – best eye** Tocilizumab (N=21)	Ocular baseline data – worst eye*** Tocilizumab (N=21)
**Other Structural Changes: Central band-keratopathy – covering visual axis**	No	28 (96.6%)	21 (100%)	20 (95.2%)
	Yes	1 (3.4%)	0 (0%)	1 (4.8%)
**Other Structural Changes: Synchiae**	No	25 (86.2%)	17 (81%)	17 (81%)
	Yes	4 (13.8%)	4 (19%)	4 (19%)
**Other Structural Changes: Iris bombe**	No	29 (100%)	21 (100%)	21 (100%)
**Other Structural Changes: Membrane formation**	No	28 (96.6%)	20 (95.2%)	20 (95.2%)
	Yes	1 (3.4%)	1 (4.8%)	1 (4.8%)
**Other Structural Changes: Neovascularisation**	No	29 (100%)	21 (100%)	21 (100%)
**Intraocular pressure (IOP)**	Mean (SD)	13.8 (3.2)	13.8 (3.5)	14.4 (3)
	Median (IQR)	13 (12, 16)	13 (11, 16)	13.5 (12.7, 16.5)
	(Min, Max)	(8, 21)	(8, 21)	(10, 21)

* Eye level – Patients that entered the trial with two eligible eyes have both eyes included in these summaries.
** Best eye – Patients that entered the trial with two eligible eyes have the best score/scenario included in these summaries at the patient level.
*** Worst eye – Patients that entered the trial with two eligible eyes have the worst score/scenario included in these summaries at the patient level.

Supplementary Table 6: Baseline rheumatoid data

Baseline rheumatoid data	Summary	Tocilizumab (N=21)
**Type of JIA (International League Against Rheumatism (ILAR) classification)**	Extended oligoarthritis	6 (28.6%)
	Persistent oligoarthritis	7 (33.3%)
	Polyarthritis RF negative	7 (33.3%)
	Psoriatic arthritis	1 (4.8%)
**Disease duration (years)**	Mean (SD)	9 (4.4)
	Median (IQR)	9.8 (5, 12.1)
	(Min, Max)	(0.5, 15.5)
**Physician global assessment of disease activity**	Mean (SD)	2 (2)
	Median (IQR)	1.6 (0.2, 3)
	(Min, Max)	(0, 7.5)
**Active joint count [all joints]**	Mean (SD)	0.9 (1.7)
	Median (IQR)	0 (0, 1)
	(Min, Max)	(0, 6)
**Swollen joint count [all joints]**	Mean (SD)	1.1 (1.9)
	Median (IQR)	0 (0, 1)
	(Min, Max)	(0, 6)
**Anti-nuclear antibody (ANA)**	Not done	1
	Negative	5 (25%)
	Positive	15 (75%)
**Double-stranded deoxyribonucleic acid (DsDNA)**	Negative	18 (85.7%)
	Positive	3 (14.3%)
**Extractable nuclear antigen (ENA)**	Not done	4
	Negative	16 (94.1%)

Baseline rheumatoid data	Summary	Tocilizumab (N=21)
	Positive	1 (5.9%)

Supplementary Table 7: Adverse Events

System Organ Class	Preferred Term	Events [Patients] (% of patients)
**Total**	**Total**	**175 [20] (95.2%)**
Blood and lymphatic system disorders	Increased tendency to bruise	1 [1] (4.8%)
	Lymphadenopathy	1 [1] (4.8%)
	Neutropenia	3 [1] (4.8%)
Congenital, familial and genetic disorders	Syringomyelia	1 [1] (4.8%)
Ear and labyrinth disorders	Ear pain	2 [1] (4.8%)
Eye disorders	Eyelid oedema	1 [1] (4.8%)
	Ocular hyperaemia	2 [2] (9.5%)
	Uveitis	3 [3] (14.3%)
	Vision blurred	3 [2] (9.5%)
	Visual acuity reduced	1 [1] (4.8%)
Gastrointestinal disorders	Abdominal discomfort	2 [2] (9.5%)
	Constipation	1 [1] (4.8%)
	Diarrhoea	2 [2] (9.5%)
	Gastrooesophageal reflux disease	1 [1] (4.8%)
	Mouth ulceration	4 [2] (9.5%)
	Oral pain	1 [1] (4.8%)
	Toothache	1 [1] (4.8%)
	Vomiting	3 [3] (14.3%)
General disorders and administration site conditions	Complication associated with device	1 [1] (4.8%)
	Injection site bruising	1 [1] (4.8%)
	Injection site reaction	24 [8] (38.1%)
	Pyrexia	1 [1] (4.8%)
Immune system disorders	Hypersensitivity	1 [1] (4.8%)
	Seasonal allergy	1 [1] (4.8%)
Infections and infestations	Cellulitis	1 [1] (4.8%)
	Cystitis	1 [1] (4.8%)
	Ear infection	1 [1] (4.8%)
	Molluscum contagiosum	1 [1] (4.8%)
	Nasopharyngitis	3 [2] (9.5%)
	Oral herpes	1 [1] (4.8%)
	Otitis externa	1 [1] (4.8%)
	Paronychia	4 [2] (9.5%)
	Skin infection	1 [1] (4.8%)

	Tonsillitis	1 [1] (4.8%)
	Tooth abscess	1 [1] (4.8%)
	Upper respiratory tract infection	3 [3] (14.3%)
	Urinary tract infection	2 [2] (9.5%)
	Viral upper respiratory tract infection	1 [1] (4.8%)
	Vulvovaginal candidiasis	1 [1] (4.8%)
	Wound infection	1 [1] (4.8%)
Injury, poisoning and procedural complications	Head injury	1 [1] (4.8%)
	Ligament sprain	2 [2] (9.5%)
	Suture related complication	1 [1] (4.8%)
	Wound	1 [1] (4.8%)
Investigations	Alanine aminotransferase increased	4 [3] (14.3%)
	Aspartate aminotransferase increased	1 [1] (4.8%)
	Blood bilirubin increased	1 [1] (4.8%)
	Blood calcium decreased	1 [1] (4.8%)
	Blood cholesterol increased	1 [1] (4.8%)
	Blood triglycerides increased	3 [3] (14.3%)
	High density lipoprotein decreased	1 [1] (4.8%)
	Intraocular pressure increased	4 [1] (4.8%)
	Low density lipoprotein increased	1 [1] (4.8%)
	Neutrophil count	1 [1] (4.8%)
	Neutrophil count decreased	4 [3] (14.3%)
	Red blood cell sedimentation rate increased	2 [2] (9.5%)
	White blood cell count decreased	1 [1] (4.8%)
Musculoskeletal and connective tissue disorders	Arthralgia	8 [4] (19.0%)
	Back pain	3 [2] (9.5%)
	Joint range of motion decreased	1 [1] (4.8%)
	Joint swelling	2 [2] (9.5%)
	Juvenile idiopathic arthritis	1 [1] (4.8%)
	Scoliosis	1 [1] (4.8%)
	Temporomandibular joint syndrome	1 [1] (4.8%)
	Tenosynovitis	1 [1] (4.8%)
Nervous system disorders	Dizziness	1 [1] (4.8%)
	Headache	8 [5] (23.8%)
	Syncope	2 [2] (9.5%)
Reproductive system and breast disorders	Dysmenorrhoea	5 [2] (9.5%)
	Hypomenorrhoea	1 [1] (4.8%)
	Menorrhagia	1 [1] (4.8%)
Respiratory, thoracic and mediastinal disorders	Cough	7 [5] (23.8%)
	Nasal congestion	1 [1] (4.8%)
	Oropharyngeal pain	7 [6] (28.6%)
	Wheezing	2 [1] (4.8%)

Skin and subcutaneous tissue disorders	Acne	1 [1] (4.8%)
	Eczema	1 [1] (4.8%)
	Erythema	1 [1] (4.8%)
	Rash	1 [1] (4.8%)
	Skin burning sensation	1 [1] (4.8%)
Surgical and medical procedures	Tooth extraction	1 [1] (4.8%)

Supplementary Table 8: Adverse Events of Special Interest

System Organ Class	Preferred Term	Events [Patients] (% of patients)
**Total**	**Total**	**21 [7] (33.3%)**
**General disorders and administration site conditions**	Injection site reaction	19 [6] (28.6%)
**Immune system disorders**	Hypersensitivity	1 [1] (4.8%)
**Skin and subcutaneous tissue disorders**	Skin burning sensation	1 [1] (4.8%)

Supplementary Table 9: Corticosteroid use – Total oral corticosteroid dose

Time period	Number registered	Received oral corticosteroids*	Total oral corticosteroid dose (mg)	Total time on study treatment – months	Total oral dose (mg) standardised to per patient year (95% CI)
**Duration of treatment period**	21	4	1683.21	68.40	295.29 (263.46, 330.97)
**Duration of treatment period and follow-up**	21	6	4458.21	129.87	411.93 (374.01, 453.69)

* Received oral corticosteroids at any point during time period.

Supplementary Table 10: Corticosteroid use – Time to reduction to 0mg

Time period	Number registered	Number included in analysis*	Number reaching 0mg	Median time to reduction (IQR)
**Duration of treatment period**	21	4	0	NA
**Duration of treatment period and follow-up**	21	4	0	NA

* Only patients that received oral corticosteroids at any point during time period were included in the analysis.
NA: Median time to reduction not reached.

Supplementary Table 11: Corticosteroid use – Time to reduction to 5mg

Time period	Number registered	Number included in analysis*	Number reaching <5mg	Median time to reduction in days (IQR)
**Duration of treatment period**	21	3	0	NA
**Duration of treatment period and follow-up**	21	3	0	NA

* Only patients that were receiving steroid eye drops at time of registration were included in the analysis.
NA: Median time to reduction not reached.

Supplementary Table 12: Steroid eye drop use – Time to reduction to <2 drops

Time period	Number registered	Number included in analysis*	Number reaching <2 drops	Median time to reduction in days (IQR)
**Duration of treatment period**	21	18	3	84 (61, 117)
**Duration of treatment period and follow-up**	21	18	5	117 (84, 140)

* Only patients that were receiving steroid eye drops at time of registration were included in the analysis.

Supplementary Table 13: Steroid eye drop use – Time to reduction to 0 drops

Time period	Number registered	Number included in analysis*	Number reaching 0 drops	Median time to reduction in days (IQR)
**Duration of treatment period**	21	20	3	61 (42, 84)
**Duration of treatment period and follow-up**	21	20	4	72.5 (51.5, 115)

* Only patients that were receiving steroid eye drops at time of registration were included in the analysis.

Supplementary Table 14: Steroid eye drop use – Post hoc-analysis – Mean number of drops

Time point	Patients included*	Mean number of drops (SD)
**Baseline**	20	4.48 (3.11)
**12 weeks**	15	4.33 (2.29)
**24 weeks**	3	3.67 (1.53)

* Only patients that were receiving steroid eye drops at time of registration were included in the analysis.

Supplementary Table 15: Visual acuity measured by age-appropriate LogMAR assessment – LogMAR Score at each time point

	Best score*				Worst score**			
**Visit**	**n**	**Missing**	**Mean (SD)**	**Median (Range)**	**n**	**Missing**	**Mean (SD)**	**Median (Range)**
**Baseline**	21	0	0.07 (0.19)	0.02 (-0.13, 0.52)	21	0	0.17 (0.32)	0.08 (-0.13, 1.20)
**4 weeks**	20	1	0.04 (0.14)	0.00 (-0.18, 0.35)	20	1	0.08 (0.17)	0.04 (-0.18, 0.40)
**8 weeks**	19	0	0.03 (0.12)	0.00 (-0.15, 0.30)	19	0	0.14 (0.34)	0.03 (-0.15, 1.40)
**12 weeks**	15	0	-0.01 (0.17)	-0.05 (-0.30, 0.40)	15	0	0.10 (0.31)	0.00 (-0.30, 0.98)
**16 weeks**	6	0	0.04 (0.08)	0.05 (-0.08, 0.12)	6	0	0.06 (0.09)	0.08 (-0.08, 0.16)
**20 weeks**	6	0	0.08 (0.06)	0.07 (0.00, 0.18)	6	0	0.12 (0.09)	0.14 (0.00, 0.22)
**24 weeks**	4	0	0.06 (0.11)	0.08 (-0.10, 0.16)	4	0	0.11 (0.15)	0.13 (-0.10, 0.26)

* Best score – Patients that entered the trial with two eligible eyes have the best LogMAR score at each visit included in the analysis at the patient level.
** Worst score – Patients that entered the trial with two eligible eyes have the worst LogMAR score at each visit included in the analysis at the patient level.

Supplementary Table 16: Visual acuity measured by age-appropriate LogMAR assessment – Joint modelling of LogMAR score and time to treatment failure

		Best score*			Worst score**		
	**Parameter**	**Estimate**	**95% CI**	**p-value**	**Estimate**	**95% CI**	**p-value**
Primary analysis	Association	-2.23	-32.96, 10.34	0.836	-4.17	-21.28, 0.41	0.457
	Time	0.02	-0.01, 0.05	0.193	0.01	-0.02, 0.04	0.332

Note: Number of observations: 70; Number of subjects: 21; Number of subjects that fail: 15 (71.4%)
* Best score – Patients that entered the trial with two eligible eyes have the best LogMAR score at each visit included in the analysis at the patient level.
** Worst score – Patients that entered the trial with two eligible eyes have the worst LogMAR score at each visit included in the analysis at the patient level.

Supplementary Table 17: Haematological and Biochemical assessment

**Analysis***	**Assessment**	**Number of abnormal clinically significant results during treatment and follow-up**
**Haematological**	**Haematocrit**	0
	**Haemoglobin**	0
	**Red blood cell count**	0
	**White blood cell count**	1 at 8 weeks
	**Neutrophils**	1 at 4 weeks<br>3 at 8 weeks<br>2 at 12 weeks
	**Lymphocytes**	0
	**Monocytes**	0
	**Basophils**	0
	**Eosinophils**	0
	**Platelet count**	0
	**Erythrocyte sedimentation rate (ESR)**	3 at 0 weeks<br>1 at 8 weeks<br>1 at 36 weeks
	**Plasma viscosity**	0
**Biochemical assessment**	**C-reactive protein**	0
	**Urea**	0
	**Creatinine**	0
	**Sodium**	0
	**Potassium**	0
	**Calcium**	1 at 20 weeks
	**Inorganic Phosphate**	0
	**Glucose**	1 at 36 weeks
	**Chloride**	0
	**Bicarbonate**	0
	**Total bilirubin**	0
	**Alanine transaminase (ALT)**	2 at 4 weeks<br>1 at 12 weeks
	**Aspartate aminotransferas (AST)**	1 at 4 weeks<br>1 at 12 weeks

* Analysed at 0, 4, 8, 12, 16, 20, 24 and 36 weeks.

Supplementary Table 18: Urinalysis test results

Analysis	Assessment	Number of abnormal results during treatment and follow-up	Number of abnormal clinically significant results during treatment and follow-up
**Urinalysis**	**Protein**	2 at 0 weeks<br>3 at 4 weeks<br>4 at 8 weeks<br>3 at 12 weeks<br>1 at 24 weeks<br>2 at 36 weeks	NA
	**Glucose**	0	NA
	**Blood**	2 at 0 weeks<br>4 at 4 weeks<br>1 at 8 weeks<br>1 at 12 weeks<br>2 at 24 weeks<br>2 at 36 weeks	NA
	**Leukocyte esterase**	3 at 0 weeks<br>4 at 4 weeks<br>1 at 8 weeks<br>3 at 12 weeks<br>2 at 24 weeks<br>4 at 36 weeks	NA
	**Specific gravity**	1 at 4 weeks<br>1 at 12 weeks	NA
	**Potential of hydrogen (pH)**	1 at 12 weeks	NA
	**Microscopic analysis**	2 at 0 weeks<br>5 at 4 weeks<br>2 at 8 weeks<br>3 at 12 weeks<br>2 at 20 weeks<br>1 at 24 weeks<br>2 at 36 weeks	1 at 24 weeks

* Analysed at 0, 4, 8, 12, 16, 20, 24 and 36 weeks.
NA: Data not collected.

Supplementary Table 19: American College of Rheumatology (ACR) Pedi response

Visit	ACR Pedi Response*	N (%)	Missing**
**4 weeks**		N=13	
	**30**	6/12 (50%)	1
	**50**	5/12 (41.7%)	1
	**70**	2/12 (16.7%)	1
	**90**	1/12 (8.3%)	1
	**100**	0/12 (0%)	1
**8 weeks**		N=12	
	**30**	7/12 (58.3%)	0
	**50**	6/12 (50%)	0
	**70**	3/12 (25%)	0
	**90**	0/12 (0%)	0
	**100**	0/12 (0%)	0
**12 weeks**		N=9	
	**30**	5/9 (55.6%)	0
	**50**	4/9 (44.4%)	0
	**70**	4/9 (44.4%)	0
	**90**	1/9 (11.1%)	0
	**100**	1/9 (11.1%)	0
**16 Weeks**		N=4	
	**30**	3/4 (75%)	0
	**50**	3/4 (75%)	0
	**70**	2/4 (50%)	0
	**90**	1/4 (25%)	0
	**100**	0/4 (0%)	0
**20 Weeks**		N=4	
	**30**	3/4 (75%)	0
	**50**	3/4 (75%)	0
	**70**	2/4 (50%)	0
	**90**	2/4 (50%)	0
	**100**	2/4 (50%)	0
**24 Weeks**		N=2	
	**30**	2/2 (100%)	0
	**50**	2/2 (100%)	0
	**70**	1/2 (50%)	0
	**90**	1/2 (50%)	0
	**100**	0/2 (0%)	0

* The ACR Paediatric 30, 50, 70, 90 and 100 levels (Giannini et al., 1997) are defined as 30%, 50%, 70%, 90% and 100% improvement respectively in a minimum of three out of 6 components in the core set with worsening of one variable by no more than 30% as defined in the ACR criteria. Patients that have 4-6 out of 6 components at baseline with the best possible score or missing data are unable to achieve an improvement at subsequent visits in a minimum of three out of 6 components so are excluded from this analysis (n=8).

** Patient has missing data at this visit so ACR Pedi Response is unable to be calculated.

Supplementary Table 20: Joint modelling of ACR data and time to treatment failure

	Parameter	Estimate	95% CI*	p-value
**ACR30**	**Association**	-1.44	-3.77, -0.31	0.003
	**Time**	-0.04	-0.62, 0.43	0.950
**ACR50**	**Association**	-1.23	-4.45, -0.13	0.006
	**Time**	0.25	-0.23, 0.72	0.296
**ACR70**	**Association**	-1.64	-5.36, 1.94	0.195
	**Time**	0.40	-0.04, 0.79	0.071
**ACR90**	**Association**	-3.03	Std.Err NaN	<0.001
	**Time**	0.88	0.22, 1.66	0.011
**ACR100**	**Association**	0.03	-0.02, 0.09	0.253
	**Time**	16.99	12.90, 23.50	<0.001

Note: Number of observations: 43; Number of subjects: 13; Number of events: 9 (69.2%)
*Credible intervals, rather than confidence intervals, due to Bayesian approach
Std.Err NaN: Standard error not a number.

Supplementary Table 21: Joint modelling of Juvenile Arthritis Disease Activity Score (JADAS) data and time to treatment failure

	JADAS10			JADAS27			JADAS71		
**Parameter**	**Estimate**	**95% CI**	**p-value**	**Estimate**	**95% CI**	**p-value**	**Estimate**	**95% CI**	**p-value**
**Association**	0.14	-0.64, 2.23	0.857	0.13	-0.63, 2.18	0.868	0.14	-0.64, 2.23	0.857
**Time**	-0.40	-2.02, 1.72	0.701	-0.36	-1.88, 1.63	0.720	-0.40	-2.02, 1.72	0.701

Note: Number of observations: 27; Number of subjects: 10; Number of subjects that fail: 7 (70.0%)
Warning: For all JADAS outcomes note low numbers of subjects/observations. Some bootstrap models took various attempts before conversion. Treat results with caution.

Supplementary Table 22: Childhood Health Questionnaire (CHQ) – Joint modelling of CHQ score and time to treatment failure

	Physical			Psychosocial		
**Parameter**	**Estimate**	**95% CI**	**p-value**	**Estimate**	**95% CI**	**p-value**
Association	-0.07	-0.31, -0.03	0.318	-0.06	-0.25, 0.10	0.546
Time	-1.55	-3.68, 0.04	0.086	0.14	-0.67, 0.82	0.695

Note: Number of observations: 63; Number of subjects: 20; Number of subjects that fail: 14 (70.0%)

Supplementary Table 23: Childhood Health Assessment Questionnaire (CHAQ) – Joint modelling of CHAQ score and time to treatment failure

Parameter	Estimate	95% CI	p-value
**Association**	3.11	1.63, 8.36	0.165
**Time**	0.01	-0.05, 0.05	0.839

Note: Number of observations: 71; Number of subjects: 21; Number of subjects that fail: 15 (71.4%)

Supplementary Table 24: Number of participants requiring change in biologic /disease-modifying anti-rheumatic drugs (DMARDS) therapy due to disease flare of their arthritis or failure to response to treatment of arthritis

Treatment	Number registered	Number requiring change in biologic / DMARDs therapy due to failure to respond from arthritis, N (%)
Tocilizumab	21	5 (23.81%)

Supplementary Table 25: Number of participants undergoing flare of arthritis (worsening of 30% or more in 3 or more of the 6 variables of the JIA core set , with no more than one variable improving by 30% or more)

**Treatment visit**		
	**Number of participants**	**Number with disease flare, N (%)**
**Overall (at least one case)**	21	0 (0%)
**Baseline**	21	0 (0%)
**4 weeks**	21	0 (0%)
**8 weeks**	19	0 (0%)
**12 Weeks**	15	0 (0%)
**16 Weeks**	6	0 (0%)
**20 Weeks**	6	0 (0%)
**24 Weeks**	4	0 (0%)

Supplementary Table 26: Number of participants in remission on and off medication of their JIA

	**Not on medication for at least 6 months, N (%)**	**On medication for at least 6 months, N (%)**	**Achieved clinical remission of JIA whilst on medication, N (%)**
**Main analysis**	3 (14.3%)	18 (85.7%)	0 (0%)
**Sensitivity analysis***	3 (14.3%)	18 (85.7%)	0 (0%)
**Additional analysis****	7 (33.3%)	14 (66.7%)	0 (0%)

* "ESR or C-reactive protein (CRP) values just outside the normal range which are not deemed to be clinically significant are nothing to be concerned with in terms of the care for the patient. Therefore, we will undertake a sensitivity analysis to classify 'abnormal but not clinically significant' ESR and CRP values as 'normal' in the definition of inactive disease of their JIA."

** "'Number of participants in remission on and off medication of their arthritis' will be analysed as per 'Number of participants in remission on and off medication of their JIA (Wallace, 2004)' with the criteria in the Wallace (2004) paper that apply to uveitis omitted to only use those that apply to arthritis. The criteria to be omitted are as follows (i) active uveitis and (ii) anti-uveitis medications."

Supplementary Table 27: Number of participants with minimum disease activity

**Treatment visit**	**Number with Oligoarticular JIA or Polyarticular JIA**	**Number with minimum disease activity, N (%)**
Overall (at least one case)	20	0 (0%)
Baseline	20	0 (0%)
4 weeks	20	0 (0%)
8 weeks	18	0 (0%)
12 weeks	14	0 (0%)
16 weeks	5	0 (0%)
20 weeks	5	0 (0%)
24 weeks	3	0 (0%)
36 weeks	0	0 (0%)

Supplementary Table 28: Foveal Thickness

	Best score				Worst score			
**Visit**	**n**	**Missing**	**Mean (SD)**	**Median (Range)**	**n**	**Missing**	**Mean (SD)**	**Median (Range)**
**Baseline**	20	1	278.6 (67.68)	257.5 (219.0,524.0)	20	1	281.7 (68.87)	262.0 (219.0,524.0)
**4 weeks**	19	2	267.4 (32.48)	267.0 (192.0,333.0)	19	2	269.0 (32.52)	271.0 (196.0,333.0)
**8 weeks**	18	1	262.9 (39.06)	265.0 (179.0,319.0)	18	1	269.6 (36.65)	269.0 (193.0,342.0)
**12 weeks**	13	2	260.9 (54.13)	270.0 (140.0,350.0)	13	2	280.9 (46.54)	286.0 (197.0,392.0)
**16 weeks**	5	1	272.2 (44.90)	293.0 (192.0,294.0)	5	1	305.6 (91.63)	294.0 (196.0,451.0)
**20 weeks**	5	1	256.8 (46.95)	271.0 (187.0,300.0)	5	1	263.0 (43.12)	280.0 (195.0,300.0)
**24 weeks**	3	1	257.7 (51.73)	285.0 (198.0,290.0)	3	1	258.3 (50.58)	285.0 (200.0,290.0)

* Best score – Patients that entered the trial with two eligible eyes have the best Foveal Thickness score at each visit included in the analysis at the patient level.

** Worst score – Patients that entered the trial with two eligible eyes have the worst Foveal Thickness score at each visit included in the analysis at the patient level.

Supplementary Table 29: Summary per participant

Improvement	Patient number	Trial status for the full 6m treatment phase	Visit used*	Days from registration date to date used	Biologics used prior to registration	MTX dose – Prior to screening	Eligible eye(s)	Eye	Drops - Baseline	Drops – 12 weeks*	AC cells score - Baseline	AC cells score – 12 weeks*	Foveal thickness - Baseline	Foveal thickness - 12 weeks*
**Responders[1]**	1	Completed 6m treatment	12 week visit	83	Infliximab[#]; Adalimumab[#]	12.5 mg/m^2	Both eyes	Right	6	2	2+	0.5+	245	197
								Left	6	2	2+	0.5+	245	196
	2	Withdrew from treatment prior to 6m treatment visit [attended 3m visit and were a response to treatment at 3m]	12 week visit	87	Infliximab	14.4 mg/m^2	Both eyes	Right	2	2	3+	0	294	288
								Left	2	2	3+	0.5+	308	240
	3	Completed 6m treatment	12 week visit	84	Adalimumab	12.6 mg/m^2	Both eyes	Right	1	1	3+	1+	267	275
								Left	1	1	3+	0.5+	272	275
	4	Withdrew from treatment prior to 6m treatment visit [committee assessed patient to be a non-response at 3m]	Unscheduled visit after 4 week visit	35	Infliximab[#]; Adalimumab[#]	13.2 mg/m^2	Right eye	Right	8	9	3+	0	524	332
	5	Withdrew from treatment prior to 6m treatment visit [committee assessed patient to be a response to treatment at 3m]	Unscheduled visit after 8 week visit	84	Adalimumab	10.3 mg/m^2	Left eye	Left	0	0	2+	0	230	238
	6	Non-response after 3m (no treatment WD completed)	12 week visit	84	Adalimumab	14.3 mg/m^2	Both eyes	Right	1	1	2+	0.5+	281	285
								Left	1	1	2+	0.5+	273	270
	7	Completed 6m treatment	12 week visit	89	Adalimumab	10 mg/m^2	Right eye	Right	2	4	4+	1+	238	297
	8	Completed 6m treatment	12 week visit	84	Adalimumab	12.5 mg/m^2	Left eye	Left	3	3	4+	0.5+	243	296
**Partial responders[2]**	1	Non-response at 3m treatment visit	12 week visit	84	Adalimumab	10.3 mg/m^2	Both eyes	Right	4	2	1+	0.5+	Unobtainable	290
								Left	4	0	1+	0.5+	Unobtainable	140
	2	Withdrew from treatment prior to 6m treatment visit [committee assessed patient to be a non-response at 3m]	Unscheduled visit after 8 week visit	76	Etanercept[#]; Adalimumab[#]	13.9 mg/m^2	Left eye	Left	1	1	1+	0.5+	238	272

	3	Non-response at 3m treatment visit	12 week visit	92	Infliximab	11.1 mg/m^2	Left eye	Left	2	2	1+	0.5+	239	286
**Non-responders[3]**	1	Withdrew from treatment prior to 6m treatment visit [committee assessed patient to be a non-response at 3m]	4 week visit	26	Adalimumab	16.6 mg/m^2	Left eye	Left	0	5	1+	3+	259	267
	2	Withdrew from treatment prior to 6m treatment visit [committee assessed patient to be a non-response at 3m]	8 week visit	56	Adalimumab	10 mg/m^2	Right eye	Right	4	12	1+	2+	219	231
	3	Non-response at 3m treatment visit	12 week visit	84	Infliximab[#]; Adalimumab[#]	10.9 mg/m^2	Right eye	Right	3	3	3+	3+	289	Unobtainable
	4	Non-response at 3m treatment visit	12 week visit	82	Infliximab[#]; Adalimumab[#]	13.9 mg/m^2	Both eyes	Right	4	4	3+	3+	380	392
								Left	4	4	2+	3+	361	350
	5	Non-response at 3m treatment visit	12 week visit	91	Adalimumab	12.8 mg/m^2	Right eye	Right	3	3	2+	2+	324	312
	6	Non-response at 3m treatment visit	12 week visit	84	Adalimumab	12.5 mg/m^2	Both eyes	Right	3	3	3+	3+	241	Unobtainable
								Left	0	0	2+	3+	247	Unobtainable
	7	Non-response at 3m treatment visit	12 week visit	91	Adalimumab	15.2 mg/m^2	Both eyes	Right	2	2	3+	3+	265	250
								Left	0	2	3+	3+	256	246
	8	Withdrew from treatment prior to 6m treatment visit [committee assessed patient to be a non-response at 3m]	Unscheduled visit after 8 week visit	53	Adalimumab	10.5 - 19.9 mg/m^2	Left eye	Left	3	6	3+	4+	313	290
	9	Non-response at 3m treatment visit	12 week visit	87	Adalimumab	10.3 - 12 mg/m^2	Left eye	Left	5	6	4+	4+	265	264
	10	Non-response at 3m treatment visit	12 week visit	80	Adalimumab	12.5 mg/m^2	Right eye	Right	0	4	2+	2+	254	220

* 12-week visit or last treatment visit if withdrew from treatment prior to 12-week visit
# Biologics used serially and not together
1 – Achieved 2-step improvement from baseline
2 – Achieved 1-step improvement from baseline only
3 – Did not achieve a 1 or 2-step improvement from baseline

Supplementary Table 30: APTITUDE Trial Management Group

Trial Management Group member	Trial Management Group role	Organisation
Professor Athimalaipet Ramanan	Chief Investigator	University Hospitals Bristol NHS Foundation Trust
Professor Michael Beresford	Chief Investigator	University of Liverpool
Professor Andrew Dick	Co-investigator	University of Bristol
Dr Catherine Guly	Co-investigator	University Hospitals Bristol NHS Foundation Trust
Dr Richard Lee	Co-investigator	University of Bristol
Katharine Wale	Sponsor Representative	University Hospitals Bristol NHS Foundation Trust
Debbie Janson	Patient and Public Involvement Representative	N/A
Dr Ashley Jones	Senior Statistician	University of Liverpool
Andrew McKay	Trial Statistician	University of Liverpool
Ben Hardwick	Supervising Trials Manager	University of Liverpool
Matthew Smyth	Trial Manager	University of Liverpool

Supplementary Table 31: APTITUDE Hospital Sites

Hospital site name	Number of patients registered
Bristol Royal Children’s Hospital	7
Great Ormond Street Hospital	6
The Great North Children’s Hospital	3
Alder Hey Children’s Hospital	2
Sheffield Children’s Hospital	2
Norfolk and Norwich University Hospital	1
Southampton Children’s Hospital	1
